# “Everyone in this together”: a qualitative study on mothers as partners in infection prevention in a neonatal unit in Botswana

**DOI:** 10.1186/s13756-026-01734-7

**Published:** 2026-03-29

**Authors:** Nidhee Jadeja, Samuel T. Matula, Ludo Magagani, Margaret Mokomane, Nina Zhu, Susan E. Coffin, Andrew P. Steenhoff, Margaret Kagiso, Britt Nakstad, Alison Holmes, Jonathan Strysko

**Affiliations:** 1 Imperial College London, London, England; 2 School of Nursing, Faculty of Health Sciences, University of Botswana, Gaborone, Botswana; 3 School of Allied Health Professions, University of Botswana, Gaborone, Botswana; 4 Division of Infectious Diseases, Children’s Hospital of Philadelphia, University of Pennsylvania, Philadelphia, PA USA; 5 Botswana Ministry of Health, Gaborone, Botswana; 6 Department of Paediatric & Adolescent Health, Faculty of Medicine, University of Botswana, Gaborone, Botswana; 7 Division of General Pediatrics, Perelman School of Medicine, Children’s Hospital of Philadelphia, University of Pennsylvania, Philadelphia, PA USA; 8 Botswana-University of Pennsylvania Partnership, Gaborone, Botswana

**Keywords:** Infection prevention and control, neonates, neonatal unit, antimicrobial resistance, mothers, qualitative study

## Abstract

**Background:**

Infection prevention and control (IPC) is critical in neonatal units to prevent healthcare-associated infections, yet research on IPC has primarily focused on healthcare workers, overlooking the role of family caregivers. In low-resource settings, mothers often take on essential caregiving responsibilities including IPC-related tasks, both a logistic necessity and a cultural norm, but their perspectives and experiences remain underexplored. This qualitative descriptive study aimed to explore mothers’ experiences and perceptions of IPC in a neonatal unit in Gaborone, Botswana. Understanding maternal involvement in IPC is essential for developing effective interventions, particularly in settings where health systems face workforce shortages and resource constraints.

**Methods:**

Fifteen semi-structured interviews were conducted with mothers of infants admitted to the neonatal unit between June and December 2022. Data were analysed using thematic analysis.

**Results:**

Six themes were identified. Major themes related to (1) mothers’ strong sense of responsibility for IPC and concerns about infection risks, (2) health system constraints (including supply shortages) that limited consistent IPC practice, (3) competing demands within restricted visiting times that required mothers to balance IPC with feeding and bonding, (4) variable communication with health care workers (HCWs) and limited comfort reminding staff about IPC, and (5) peer dynamics between mothers, including mutual reminders as well as tensions arising from shared feeding equipment. A minor theme that emerged was (6) mothers’ emotional distress and its perceived impact on remembering and enacting IPC behaviours. Overall, mothers described high motivation to protect their infants but reported that time pressures, insufficient communication with HCWs, and resource limitations constrained IPC adherence, underscoring the need for structured IPC orientation and ongoing support for families in neonatal care.

**Conclusion:**

Mothers in this neonatal unit viewed themselves as key partners in IPC and were highly motivated to protect their infants, but their ability to consistently follow IPC practices was constrained by limited supplies, staffing pressures, restricted time for caregiving and bonding, and variable communication with HCWs. Mothers also expressed concerns about HCW adherence to IPC and described how emotional distress and peer dynamics could shape IPC behaviour. These findings underscore the need for structured IPC education and support for mothers in neonatal units, and the importance of integrating family-centred IPC policies into neonatal care. Future research should evaluate interventions that strengthen caregiver support, improve communication, and address contextual barriers and constraints to sustained IPC practice.

**Supplementary Information:**

The online version contains supplementary material available at 10.1186/s13756-026-01734-7.

## Background

Neonatal sepsis is a leading cause of under-five mortality globally, accounting for 400,000 to 900,000 deaths annually [1]. Adherence to infection prevention and control (IPC) standards in hospital neonatal units (NNUs) is critical to prevent healthcare-associated sepsis in hospitalized neonates, who are at heightened risk of infection due to their underdeveloped immune systems, extended hospital stays, and frequent need for invasive procedures. In low-resource settings, the challenge of maintaining effective IPC is exacerbated by system-level constraints such as limited healthcare personnel, inadequate infrastructure, crowding and shortages of essential supplies [2, 3].

Traditionally, hospital IPC programs have focused on the role of health care workers (HCWs) - nurses, doctors, and allied health professionals - often overlooking the contribution families can make to IPC efforts [4]. However, family-centred care is increasingly acknowledged as important, encouraging families to be involved in the care of their babies to promote bonding and be active partners in the treatment and care journey [5, 6]. The global HCW shortage, most acute in low- and middle-income countries (LMICs) has led to task-shifting, where mothers in resource-limited NNUs assume essential patient care duties such as feeding, bathing, and even administering medications [7–10].

Despite a number of qualitative studies that explore the psychosocial stressors faced by families of hospitalized infants, there remains a significant gap in understanding mothers’ perspectives on IPC in LMICs [11]. Understanding their views is essential for designing effective interventions that engage families in IPC, an approach that shows promise in resource-constrained environments [12].

This qualitative study aimed to explore mothers’ experiences and perceptions of IPC in the NNU of a referral hospital in Botswana. Specifically, through in-depth interviews and thematic analysis, this study aimed to: (1) describe mothers’ understanding of IPC measures relevant to the care of their infant, (2) explore how mothers perceived their own responsibilities for IPC and those of HCWs, (3) examine how communication with HCWs and other mothers shaped IPC-related behaviour; and (4) identify structural and organisational barriers and enablers affecting mothers; participation in IPC. Building on evidence that family caregivers in low-resource settings often undertake essential caregiving tasks with limited formal training, and that communication and power dynamics can shape caregiver participation, it was anticipated that these themes would emerge in mothers’ accounts [4, 13–16]. The study also explored whether mothers perceived stress and emotional burden to influence their capacity to follow IPC practices as an emergent question during analysis.

## Methods

### Study setting

 This study was conducted at a 530-bed public referral hospital serving Botswana’s capital city and surrounding region. The Neonatal Unit (NNU) is a tertiary care facility with 35 beds and admits approximately 100 newborns every month.

### Design

 A qualitative descriptive study was conducted using semi-structured interviews between June to December 2022. This approach enabled an in-depth exploration of mothers’ experiences and perceptions of IPC in the NNU. WHO guidance on the core components for IPC in health facilities provided the foundational framework for the interview guide. The sample size aimed to achieve thematic saturation [17].

### Participant selection and consent

 Ward staff informed mothers about the study. The research team then provided a brief verbal overview to mothers during times when they were not attending to their infants and invited interested mothers to speak with the team in a designated private interview room. As participation was individual opt-in, the number of mothers who heard about the study but chose not to participate was not formally recorded; no mothers who presented to the interview room declined participation, and no participants withdrew after consenting. Eligible participants were mothers aged 18 years or older with a baby admitted to the NNU during the study period. Participation was voluntary, with mothers given an information sheet in both English and Setswana, and at least 30 min to decide before providing informed consent. A small cash reimbursement of P30.00 was offered to mothers as recommended by the Ministry of Health and Wellbeing IRB. Given it is a sensitive and worrying time for mothers with their baby requiring hospitalised care, interviewers were trained to engage participants with concern, respect, equity, allowing free will, and without insensitivity or prejudice [18].

### Data collection and translation

 Interviews were conducted in Setswana or English, based on participant preference, and audio-recorded. All interviews were conducted in a private space at the hospital, near the NNU but away from clinical care areas to support confidentiality. The interviews ranged from 15 min to 40 min in duration and were audio-recorded with permission. No repeat interviews were conducted. Interviews were conducted by two members of the research team and no HCWs were present during the interviews. NJ, Ph.D researcher with a background in public health, conducted all interviews in English, while LM, a research nurse, fluent in Setswana and English, conducted all interviews in Setswana. Both interviewers identify as female, and were trained in qualitative interviewing techniques prior to the study. Neither interviewer was a member of the neonatal unit clinical team, or had a prior clinical, supervisory, or managerial relationship with participants. Using two interviewers enabled interviews to be conducted in the preferred language of participants while maintaining a consistent interview approach. NJ and LM held brief debriefs during data collection and reviewed emerging topics to ensure similar prompting across English and Setswana interviews.

Setswana interviews were transcribed verbatim in Setswana and then translated into English by LM, under the supervision of SM, who are both native Setswana speakers, with discrepancies resolved through discussion between LM and SM. Translation happened at three stages of the study: the development of the semi-structured interview guides, information sheets, and consent forms; the data collection process which involved conducting the interviews; and the analysis phase, which required translating transcribed Setswana interviews into English.

### Data analysis

Transcripts were analysed by NJ using thematic analysis, combining deductive coding (informed by WHO IPC core components and prior literature on caregiver roles in LMIC neonatal care) with indicative coding to capture unexpected or context-specific issues [19]. An initial coding framework was developed by NJ after familiarisation with the dataset and iteratively refined as coding progressed. JS, SM, and AH independently reviewed identified codes and categories. Two peer Ph.D researchers at Imperial College independently coded 3 transcripts each and differences were discussed to refine code definitions and ensure consistent application. NVivo software was used for organisation and coding, with detailed notes recorded through the analysis process.

Codes were grouped into candidate themes and reviewed through discussions with the research team. An initial set of themes, such as mothers’ recognition of the importance of IPC, significant structural and resource limitations, and ward-level socio-cultural and power dynamics, were anticipated based on existing literature on the role of mothers in IPC in neonatal care in LMICs [13–16, 20]. Other themes emerged inductively through repeated engagement with the data.

### Ethical considerations and Confidentiality

 Participants were informed of their right to withdraw from the study at any time. Interview transcripts were de-identified, and stored securely in a password-protected online system, accessible only to the study team. Ethics approval was obtained from the University of Botswana, the Hospital, the Ministry of Health Botswana, and the Head of Department of Infectious Disease and Research Governance and Integrity Team (RGIT) at Imperial College London.

Signed consent forms, audio-recordings, and transcripts were accessible only to the study team to safeguard participants’ comfort and safety. Concerns regarding safety or quality of care raised during interviews were reported anonymously to a study team member based within the NNU. Participants were informed of this feedback mechanism to provide reassurance.

This study followed the Standards for Reporting Qualitative Research and the Consolidated Criteria for Reporting Qualitative Research (COREQ) checklist to ensure adherence to established qualitative research reporting standards [21, 22].

## Result

15 interviews were conducted with mothers, 10 of them in Setswana and 5 in English. The median age for interviewed mothers was 28 (range 20–42) and mean parity was 2 (range 1–9). Most infants were premature with a median gestational age at birth of 28 weeks (range 23–42)).

Themes were defined as patterned and conceptually coherent issues that meaningfully captured mothers’ accounts of IPC-related experiences. Major themes were those discussed by mothers in at least 8 of the 15 interviews, and minor themes as those discussed in fewer than 8 interviews.

Five major themes and one minor theme emerged from the interviews with mothers:

1. Sense of responsibility and concern about infection control.
2. Mothers’ experience of system constraints on infection prevention and control.
3. Balancing demands and time pressures on IPC.
4. Mothers’ relationships with healthcare workers relating to infection prevention and control.
5. Mothers’ relationships with each other relating to infection prevention and control.
6. Emotional strain and its perceived impact on IPC practice (minor).

Table 1 below provides a summary of these themes and associated sub-themes, which are presented in detail in this section.

**Table 1:** Themes and sub-themes emerging from interviews (major themes discussed in at least 8 of 15 interviews; minor themes as those discussed in fewer than 8 interviews)

Theme	Sub-theme
1. Sense of responsibility and concern about infection control	1.1 ‘I take accountability’: Mothers’ strong sense of responsibility and ownership for infection prevention and control
	1.2 Maternal concerns regarding HCWs as potential sources of infection transmission
2. Mothers experience of system constraints and impact on IPC	2.1 Supplies
	2.2 Workforce
3. Balancing demands and time pressures on IPC	
4. ‘It depends on who you’re talking to’: Perceptions and relationship with HCWs on IPC	4.1 Communication with HCWs on infection prevention and control
	4.2 Reminding HCWs on infection prevention and control practice
5. ‘Everyone in this together’: perceptions and relationship between mothers relating to IPC behaviour	5.1 Mothers’ perceptions of peer hand hygiene practices
	5.2 Reminders and receptivity: peer influence among mothers on infection prevention and control
	5.3 Shared breastfeeding utensils as a source of infection concern for mothers
6. Emotional strain and its perceived impact on IPC practice (minor)	6.1 Witnessing infection-related illness and death heightened IPC vigilance and fear
	6.2 Distress and cognitive overload were perceived to reduce adherence to IPC routines

## Sense of responsibility and concern about infection control

Mothers voiced a strong sense of responsibility and ownership relating to infection prevention and doing everything they could to protect their baby from infection. They also expressed concerns regarding HCWs handling their babies and the potential risks of infection when hand hygiene was not followed. They also in turn highlight the importance of trust, practicing IPC compliant behaviour, and communication between HCWs and mothers to address their concerns.

### “I take accountability”: Mothers’ strong sense of responsibility and ownership for IPC

The understanding from mothers that the stakes are high for their very vulnerable babies was acute, and they conveyed a strong sense of personal responsibility for IPC, conversely twinned with great worry about their baby contracting an infection. The excerpts below highlight this:

*“To reduce the spread of infections and also be certain that my child is safe and that there is a low risk of infection. I take accountability to make sure that my baby is safe.” –* ***Mother 1***, **translated from Setswana**.

*“I want to be comfortable and free knowing that when a person enters the room I am able to tell them to do this and don’t do that*,* like being in control knowing that I am also protecting my baby making sure that where I am is clean and safe.” –* ***Mother 8***, **translated from Setswana**.

Despite feeling alone, these quotes overall demonstrate that mothers are not passive, and are proactive and seek to assert their control in protecting their babies.

### Maternal concerns regarding HCWs as potential sources of infection transmission

Mothers expressed concerns regarding potential sources of infection transmission within the NNU, particularly regarding HCWs and specifically nurses. They observed when HCWs did not consistently practice hand hygiene or IPC measures when handling infants, and expressed their worry that infections may be transmitted.

*“We are many*,* we come from different wards and we don’t know each other. What worries me is that the cleaners enter and the nurses enter*,* I am not sure if this person is ok or not ok in terms of whether they will bring an infection with them.”* ***– Mother 5***, **translated from Setswana**.

*“I feel that nurses are also carriers of infection because they touch here and there and it is not all of them who wash their hands you see*,* and also it is not all of them who wear gloves when touching a baby who has an infection. After they touch the baby with the infection they go on to touch the baby without the infection*,* I think they are carriers of infection apart from us parents. They feel that we don’t wash our hands properly but they also contribute to infections in the babies.”* ***– Mother 3***, **translated from Setswana**.

*“Yes*,* sometimes they use gloves then from there they go straight to touch another baby*,* they can also touch them with bare hands you see. So that also contributes to infection in the babies. The nurses just feel that it is us parents who bring infections you see. We as parents are very careful since we don’t want our babies getting the infections.”* ***– Mother 13***, **translated from Setswana**.

The last two quotes reveal the mothers’ perception of experiencing a sense of blame from HCWs, prompting them to respond by redirecting this sentiment towards the nurses and doctors. Their perception that nurses placed the blame solely on mothers suggests a need for improved communication on a shared responsibility approach between HCWs and mothers in IPC.

## Mothers experience of systems constraints and impact on infection prevention and control

Mothers described at length the repercussions of system constraints and shortcomings in IPC, namely supply shortages, and workforce constraints.

### Supplies

Mothers highlighted their concerns with shared feeding utensils and the limited supply of feeding tubes:

*“It is sharing the baby’s feeding utensils with other mothers*,* we all share the feeding utensils and no one has their own feeding utensils. Like for example there would be four feeding dishes*,* but there are twenty mothers that need to feed their babies. When I leave here to go see the baby…since we go at different times*,* the 9 o’ clock group uses the feeding utensils and when another group gets there at 10 o’ clock they use those feeding utensils so you would not know if they are thoroughly clean.”* ***– Mother 7***, **translated from Setswana**.

*“If you look closely inside the feeding tube you will see milk that looks like it has gone bad. So it needs to be changed time and again*,* a baby should not be kept for days with it*,* a baby should not be kept for long with it because when it is like that*,* the new milk that I come with when I put it inside the tube it will then push that old milk that looks like it has gone bad*,* pushing it where? Inside the baby’s stomach and then the baby starts to experience diarrhoea.”* ***– Mother 8***, **translated from Setswana**.

### Workforce

Some mothers observed that HCWs were short-staffed and under pressure, and felt that the appropriate care for their babies suffered as a result.

*“What I am not happy about is that a nurse would be working alone but the babies are over 20*,* you cannot give only one baby attention… that is dangerous for these babies.”* ***– Mother 3***, **translated from Setswana**.

The mothers observed that nurses were busy and rushed and in attending to critical situations often did not have the time to practice hand hygiene or the changing of gloves between patients. Mothers conveyed a sense of understanding the pressures HCWs faced, but also recognised the link between short-staffing and compromises to patient care and infection control.

## Balancing demands and time pressures on IPC

Mothers had one hour every three hours with their baby, as per NNU policy to manage overcrowding, in which they were to express milk and feed their babies, clean their babies and cot, and perform Kangaroo Mother Care and bonding. Mothers described a pervasive sense of pressure during their time with their babies and the impact of this on remembering and prioritising hand hygiene and other tasks. The excerpt below exemplifies the balance of essential caregiving tasks mothers expressed they were trying to navigate:

*“If we were given longer time*,* when we get there*,* we would just take our time making sure that you have washed your hands thoroughly. From here I do everything in a rush because I have to take 30 minutes kangarooing my baby because that is what I enjoy then these other things I would just do them in a hurry so that I can have more time to kangaroo my baby. So if we were given more time*,* these things they are easier to follow*,* it’s very easy to keep your environment clean*,* to wash the things that you use*,* to wash your hands.”* ***– Mother 5***, **translated from Setswana**.

This above quote speaks not only to the issue of a sense of competing priorities for the mother, but also to the aspect of easily accessible handwashing sinks and hand alcohol gel.

## “It depends on who you’re talking to”: Perceptions and relationship with HCWs on infection prevention and control

 Mothers reported a wide range of experiences relating to communication with HCWs. As described above, some mothers felt communication with HCWs was rushed, but they also described their experience communicating on IPC with HCWs, and their comfort level reminding HCWs on IPC practice. Overall, mothers felt more comfortable asking for supplies they needed (e.g. more soap), and less comfortable asking questions for more information or reminding HCWs on infection control practice.

### Communication with HCWs on infection prevention and control

*“They’re kind of impatient*,* and then there are ones that are just generally great people like Dr X*,* you don’t even have to ask her questions. She is just dishing out the information.”* ***– Mother 1***.

*“It depends who you’re talking to.”* ***– Mother 11***.

*“You don’t even know what infection is he having? Is it something that will pass through the air or what? They are just vague. Should I be worried or not?”* ***– Mother 4***.

*“They give a little time. So you won’t have time to ask a lot of questions. They’ll just say kangarooing is best for the baby*,* do kangaroo. So I have to find out what is kangaroo? What does it do to the baby?”* ***– Mother 15***, **translated from Setswana**.

The last quote shares how important it is for use of terminology such as ‘kangaroo care’ to be clarified as part of the orientation for mothers on their meaning and what it entails. All these quotes demonstrate the varied experiences mother had with different HCWs. Mothers experienced frustration when they lacked information and clarity on their infant’s health and progress, and frustration with the limited time available to engage with HCWs to ask questions.

### Reminding HCWs on infection prevention and control practice

*“I don’t want to offend anyone.”* ***– Mother 11***, **translated from Setswana**.

*“This is your child’s healthcare and if I don’t feel comfortable with you not following protocol even though you’re the one who supposedly been taking care of my child*,* I should have a say*,* to say*,* hey?”* ***– Mother 6***.

*“Some of them we are not comfortable to say anything to them because they are always having that anger. You already know that this one if you talk to them they might shout at you.”* ***– Mother 1***, **translated from Setswana**.

*“We are scared of them.”* ***– Mother 5***.

*“The hospital staff tell us if we not doing things properly but for us we just look at them without saying anything when they don’t do things properly. I am scared to talk in such instances.”* ***– Mother 1***, **translated from Setswana**.

These excerpts provide an insight into the dynamics of communication on IPC when mothers were asked how comfortable they feel asking questions, reminding, or raising concerns regarding IPC practice with the HCWs. Mothers felt they had to balance asserting their responsibility and right as parents with not wanting to offend the HCWs and eliciting a negative reaction from them. A few mothers expressed an element of reminding or raising concerns on IPC as overstepping their boundaries as a mother and caregiver, which indicates the power dynamics and hierarchies at play. A few mothers suggested that nurses responded defensively when approached on something the mother felt was not being done right or needed addressing, which could potentially be seen as dismissing the mother’s input and may further discourage open communication.

## “Everyone in this together”: perceptions and relationship between mothers relating to infection prevention and control behaviour

 Most mothers discussed mutual willingness to share IPC information and felt comfortable reminding each other. Some mothers did not feel equally as comfortable, and a particular area of concern revolved around the shared utensils for breastfeeding. This issue was expressed as a potential source of friction between mothers, and they felt it could threaten the overall sense of harmony between them. Mothers rated the IPC practices of each other as lower than their own standards. They felt some mothers were not as vigilant in maintaining cleanliness with shared breastfeeding utensils and hand hygiene after using the toilet as they personally were. Mothers acknowledged that nurses and support staff were aware of the cleanliness and disinfection challenges associated with the shared breastfeeding equipment and were often reminding them.

### Mothers’ perceptions of peer hand hygiene practices

*“Other mothers are careless*,* they are really careless and I would sometimes say to myself that ‘eish! If only this baby was mine’.”* ***– Mother 3***, **translated from Setswana**.

*“Some people you honestly cannot remind them*,* they will ask you ‘why you are looking at me.’ That is the response they give you or someone just starts to dislike you because of asking a simple question ‘why are you not washing your hands?’”* ***– Mother 2***, **translated from Setswana**.

In these instances, there is an element of mothers judging one another as not understanding their responsibilities and the vulnerabilities of their babies. In the first quote in particular the mother is applying blame to the mother for their infants being sick.

### Reminders and receptivity: peer influence among mothers on infection prevention and control

Mothers discussed peer influence on IPC in two ways: sharing information and insight they had learned, and reminding each other. This demonstrates that mothers actively contributed to the reinforcement of IPC practices within the mothers´ network and nurtured a supportive environment for exchanging information and encouraging each other on IPC.

*“Yes we do talk and share what we have been taught such as this is what I learnt or this is what the doctor told me. From all that you learn something.”* ***– Mother 4***, **translated from Setswana**.

*“We do share a lot. Everyone in this together.”* ***– Mother 13***, **translated from Setswana**.

*“That is why I said that we teach each other as mothers*,* we remind each other. If you are stressed you can tell the next mother that I am stressed I don’t feel like feeding my baby and you guys would just talk freely and it would feel like you are at home.”* ***– Mother 2***, **translated from Setswana**.

### Shared breastfeeding utensils as a source of infection concern for mothers

*“We should not share*,* everyone should have their own utensils knowing that this is for my baby only. I would use them and I would know that I have thoroughly washed them. Knowing that I handle these things properly because my baby’s life depends on them but we share them and you will never know another person’s intentions. Another mother does not care if the syringe has been thoroughly washed and even if you call her and tell her ‘look at the syringe that you were using’*,* it ends up causing friction. It is like you are being difficult person.”* ***– Mother 5***.

*“We share the breastfeeding equipment*,* so we wash things in different ways. As we exchange them*,* you don’t know if someone will also wash those utensils 100% thoroughly.”* ***– Mother 6***, **translated from Setswana**.

These quotes highlight the level of concern with the level of cleanliness observed among mothers with shared breastfeeding utensils and their potential as a source of friction between mothers. In the last quote the mother re-washed utensils she felt were still dirty after the disinfection process, before she used them on her infant but what she didn’t realise is that this can reintroduce the potential for infection as the sinks and tap water are not sterile. This is an example of the intricate details and nuances of IPC that are required, and therefore educating and reinforcing these nuances to mothers becomes crucial.

## Emotional strain and its perceived impact on IPC practice (minor theme)

*“It is an on and off thing*,* so we cannot say ‘we are fine’ or anything you see.” –* ***Mother 3***, **translated from Setswana**.

Although mothers were not explicitly asked to provide descriptions of their mental health or emotions, they often reflected on their mental well-being during discussions about infection prevention. They described the significance of maternal mental health in relation to infection prevention considerations as well as the highly stressful and sensitive circumstances in which mothers found themselves. Their narratives revealed experiences of anxiety, fear, and trauma as they observed what was happening in their environment, and the potential risks and impacts of infection for both other infants and their own baby. They expressed a sense of lacking emotional support and finally, alluded to the impact of their mental health on their infant, as well as their capacity to remember and practice infection control protocols. These aspects are described below.

### Witnessing infection-related illness and death heightened IPC vigilance and fear

*“The first time I came*,* he was in ICU and I see a lot of those kids with critical situations. So I had to see some dying. So it was traumatising for me. I was like*,* what is going to happen with my baby? Your baby is with kids who have infections.” –* ***Mother 15***, **translated from Setswana**.

*“A lot of babies were dying last week*,* this week. In a day*,* five babies would die whilst we´re still here and this really frustrated us. We were always crying each and everyday*,* hearing that five babies died in a day as a result of an infection*,* sepsis and other diseases.” –* ***Mother 6***.

These excerpts reveal the emotional trauma experienced by mothers in witnessing critical situations in the NNU, as well as their concerns about infections and the impact on their own infants. The presence of other infants with infections created anxiety and uncertainty, compounded by the lack of information they felt they were receiving on their baby.

### Distress and cognitive overload were perceived to reduce adherence to IPC routines

*“We are not happy to be in this environment*,* there is confusion sometimes you see*,* not because we are forgetful but because the mind would be elsewhere and having countless thoughts.” –* ***Mother 4***.

*“Yeah*,* it’s been very difficult. Sometimes you’ll find you just cry and you don’t know why am I crying? Why am I this sad? But you have to avoid those feelings because it’s not good for the baby.” –* ***Mother 15***, **translated from Setswana**.

Mothers frequently conveyed feelings of isolation, exhaustion, a feeling of lack of support, and overall depression and hopelessness regarding their circumstances. These accounts suggest mothers understood IPC as important yet perceived that emotional distress and cognitive load reduced their ability to consistently enact IPC behaviours for their infant, an effect that warrants attention in IPC interventions that rely on caregiver participation.

## Discussion

Mothers play a crucial role in infection prevention, not just through breastfeeding and kangaroo mother care, but also in caregiving duties where adherence to IPC standards is vital. However, in this study, mothers described shouldering these responsibilities in isolation and under significant stress. There is growing recognition of the need to formalise the involvement of family caregivers in IPC in LMICs [4, 23]. A study investigating the inclusion of patients and family members in IPC across LMIC settings found that family members’ caregiving contributions were comparable to those provided by HCWs in higher-income countries, yet they often lack the formal training or education [4, 24]. Remarkably, research on the caregiver experience within healthcare settings remains relatively scarce, particularly for mothers of hospitalised neonates in LMIC contexts.

A recent study in Botswana found that mothers of newborns admitted to the NNU experience stress particularly in relation to parental role adjustment and HCW communication [25]. Evidence from neonatal units in Malawi and Ghana show that mothers have a good understanding of IPC principles but face substantial structural and organisational constraints that limit their ability to enact best practice, particularly in settings characterised by overcrowding, resource scarcity, and limited communication with healthcare workers [13–15, 20]. Evidence from Ghana and Zimbabwe highlight how ward-level socio-cultural dynamics, especially hierarchical relationships between healthcare workers and mothers shape communication, constrain caregiver participation, and reduce the priority given to IPC as a shared responsibility [13, 14, 16, 20]. Our findings resonate strongly with this literature, while extending it by shedding new light on their mental health experiences, time pressures, and peer dynamics. Together, this emerging evidence suggests that interventions designed to improve communication, reduce the sense of hierarchical barriers, and provide structured IPC orientation and readily available access to information for families, are likely to be transferable to other resource-constrained neonatal settings facing similar systematic challenges.

This setting offers a valuable perspective on neonatal outcomes and IPC practices with transferable relevance to lower-income settings where efforts are underway to shift more births to healthcare facilities. Despite progress in antenatal care and facility-based deliveries, Botswana’s persistently high infant mortality rate of 28 per 1000 live births underscores the disconnect between facility births and improved outcomes [26]. For example, in 2017, 97.1% of bloodstream infections among hospitalized neonates were hospital-acquired (> 72 h after admission) [27]. In 2018, a prospective observational cohort study in the same hospital found a high burden of infection, with 61.1% of infants diagnosed with sepsis at least once during their admission [28–30].

Recent research highlights critical IPC gaps in Botswana’s hospitals. A 2019 prevalence survey in a tertiary referral hospital found over 10% of hospitalised patients acquired infections, with higher risks in ICUs [31]. In a retrospective cross-sectional study at a private hospital in Gaborone, among 77 cases of *A. baumannii* pneumonia from 2011 to 2016, more than 98% exhibited either multi-drug resistance, extensive drug resistance, or pan-drug resistance [32]. A recent whole genome sequencing study on *A. baumannii* isolates from patients and the NNU environment revealed spatial clustering in NNU sinks [33]. Lastly, the Covid-19 pandemic further exposed IPC vulnerabilities, with documented hospital transmission of SARS-CoV-2 reported among patients, caregivers, and healthcare workers in the NNU [34].

Findings from this study have led to direct changes in hospital practice, including an overhaul of feeding utensil cleaning methods, elimination of shared feeding syringes, and developing an IPC-focused orientation for families. These actions highlight the importance of capturing and elevating maternal perspectives into NNU improvements.

## Limitations

The first limitation of this study is the challenge of generalisability given the single-setting context. Efforts were made to mitigate this limitation in generalisability through detailed documentation of the research process and expert consultations on the applicability of emergent findings to other settings. While some findings may not be universally applicable, they offer valuable insights and a foundation for further research in similar low-resource healthcare environments.

Language differences presented a challenge. Participants could choose English or Setswana, but some non-native English speakers may have struggled to fully express ideas. Similarly, for those interviews conducted in Setswana, it is possible that certain concepts may not have been fully translatable, as translation is also an interpretive process and nuances may get lost, affecting the accuracy of the collected information [35]. Additionally, some participants may have spoken other first languages, such as Kalanga, which could have influenced comfort levels, though no concerns were raised. This may have affected nuance in mothers’ descriptions of emotions, relationships, and IPC practices.

The interviews were conducted by researchers with clinical and academic backgrounds in public health and infection control. The gender of the interviewers, which was the same as that of participants, may have facilitated rapport and encouraged discussion of caregiving experiences and emotional wellbeing. However, participants may have also perceived interviewers as aligned with the clinical team, potentially influencing the extent to which they felt comfortable critiquing health practices or staff. Although efforts were made to minimise power imbalances through reassurance of confidentiality and a non-judgemental interview approach, interviewer positionality remains a potential source of bias.

Finally, conducting interviews within the hospital setting may have influenced the willingness of mothers to criticise institutional practices. Efforts were made to mitigate this by conducting interviews in private spaces and emphasising confidentiality.

## Conclusion

Mothers are critical yet often overlooked stakeholders in neonatal IPC, bearing significant caregiving responsibilities despite limited formal training or support. This study highlights the intersection of health system constraints, maternal mental wellbeing, and IPC adherence. The findings underscore the need for structured, family-inclusive IPC interventions in LMIC health facilities. Strengthening mother-HCW communication, peer support networks, and culturally tailored IPC education is important. Future research is required to evaluate sustainable approaches to meaningfully integrate mothers into IPC efforts, ensuring policies reflect their lived realities in resource-limited settings.

## Supplementary Information

Below is the link to the electronic supplementary material.

Supplementary Material 1

## Data Availability

The datasets generated and analysed for this study are not publicly available due to the sensitive and potentially identifiable nature of the qualitative data, but are available from the corresponding author on reasonable request, subject to ethical approvals and data-sharing agreements.

## Abbreviations

AMR: Antimicrobial Resistance
AMS: Antimicrobial Stewardship
HAI: Hospital Acquired Infections
IPC: Infection Prevention and Control
LMIC: Low- and Middle-Income Country
NNU: Neonatal Unit
WHO: World Health Organization
