# Supplementary material for: “Everyone in this together”: a qualitative study on mothers as partners in infection prevention in a neonatal unit in Botswana
Source: Antimicrob Resist Infect Control. 2026 Mar 29;15:68. doi: 10.1186/s13756-026-01734-7 (PMC13151290; doi:10.1186/s13756-026-01734-7)
Supplement: Supplementary file 1 — Supplementary Material 1 [file 13756_2026_1734_MOESM1_ESM.docx]

**Semi-Structured Interview Guide – mothers**

**Introduction**

Please could you tell me a bit about yourself:

1. Your age
2. Can you tell me about yourself and your family?
3. How many pregnancies? How many live births?
4. Could you please tell me about your baby?

*Probe: When and where was the baby born? How long your baby has been in hospital? For what reason?*

1. Could you please describe how your day looks like?

*Probe: How much time you spend here in the hospital on a daily basis, and how much time with your baby directly? What influences how much time you have with your baby? (probe ie hospital rules) What are the things you do with your baby when you are together in hospital (probe: kangaroo care, breastfeeding, etc) When not with the baby what do you do?*

**[Individual-level factors influencing IPC]**

1. In addition obviously to your baby’s condition, is there anything else you are worried about in terms of your baby’s stay here?

*Probe: infection?*

1. Do you know what practices can help prevent spread of infection among neonates while in hospital like this?

*Probe: hand hygiene, preparation or storage of breastmilk/formula*

1. Do you observe these practices yourself in care of your neonate?

*Probe: what makes it easy to practice those? What makes it difficult to practice them? Which ones do you usually perform?*

1. How confident do you feel about them?

*Probe: which ones do you feel confident about? Which ones do you not feel confident about? What can be done to help you be more confident with them*?

1. Do you observe these practices among other mothers?

*Probe: If they do not practice what usually happens? What do you expect to happen? Who is supposed to ensure that mothers do this?*

**[Organisational-level factors influencing IPC]**

1. Do you observe these practices among the hospital staff?

*Probe: at all times? Do you feel comfortable saying anything? Do you think mothers should say anything?*

1. Have you ever been provided with information about helping to prevent infection in your baby while hospitalised?

*Probe: if so, what did you receive? How did you receive this? Where have you seen this? What format was it in? Can I see it? If not, what information would you like? And how?*

1. Do you feel there is sufficient access and availability of handwashing sinks and soap for you to keep your hands clean?
2. Do you feel the environment in the neonatal ward is clean?
3. Do you feel the environment in the neonatal ward is crowded?
4. How would you describe communication between mothers on infection prevention? *Probe: do they share information about infection prevention? Do they ask each other questions instead of HCW?*
5. How would you describe communication between you and the HCWs?

*Probe: do you feel HCWs give you time and support in terms of best care-related practices to reduce the risk of infection for your baby? Can you ask them questions? Do you feel you can trust them?*

1. What are the sources of information for you on how to best care for your baby?

*Probe: friends, family, whatsapp, social media?*

1. Who would you say has the strongest influence?

**Conclusion**

1. Is there anything else you’d like to tell us that we haven’t asked about?
